# Supplementary material for: Physical activity in adult users of inpatient mental health services: A scoping review
Source: PLoS One. 2024 Aug 19;19(8):e0301857. doi: 10.1371/journal.pone.0301857 (PMC11332955; doi:10.1371/journal.pone.0301857)
Supplement: S2 File — (DOCX) [file pone.0301857.s002.docx]

**PIPA data extraction form**

| **Scoping review details** | |
| --- | --- |
| Scoping review title | Physical activity in adult users of inpatient mental health services: a scoping review |
| Review objective | To understand the extent and type of evidence in relation to physical activity in adult users of inpatient mental health services |
| Review questions | 1. What are the characteristics of physical activity intervention studies for this population?  2. What correlates of physical activity have been explored or reported in adult users of inpatient mental health services? |
| **Inclusion/exclusion criteria** | |
| Participants/context | We will include studies pertaining to adult (≥18 years) users of inpatient mental health services. This would include both studies where psychiatric inpatients are the participants and studies where other stakeholders are involved (e.g., healthcare professionals giving their views regarding services for psychiatric inpatients). Studies focusing on learning disability populations will be included whereas those focusing on eating disorder populations will be excluded. There will be no limits on the country of origin. |
| Concept | The phenomenon of interest is physical activity. Physical activity is defined as any bodily movement produced by skeletal muscles and requiring energy expenditure. Exercise is a subset of physical activity that is defined as any structured and repetitive physical activity that has an objective of improving or maintaining physical fitness. We will include studies that address the aforementioned review questions and will take a broad view of physical activity, including studies that have focused on supervised exercise or promotion of self-managed physical activity. We will exclude studies on physical activity that do not relate to the review questions. |
| Types of evidence source | This scoping review will consider both experimental and quasi-experimental study designs including randomised controlled trials, non-randomised controlled trials, before and after studies and interrupted time-series studies. In addition, analytical observational studies including prospective and retrospective cohort studies, case-control studies and analytical cross-sectional studies will be considered for inclusion. This review will also consider descriptive observational study designs including case series, individual case reports and descriptive cross-sectional studies for inclusion. Qualitative studies will also be considered that focus on qualitative data including, but not limited to, designs such as phenomenology, grounded theory, ethnography, qualitative description, action research and feminist research. Review articles and meta-analyses will be used to identify primary studies, but will be excluded from data analysis. Conference abstracts and opinion papers will be excluded. |
| **Evidence source data** | |
| Citation details (e.g. author/s, date, title, journal, volume, issue, pages) |  |
| Country |  |
| Aim(s)/purpose? |  |
| Relates to which scoping research question(s)? |  |
| Setting (e.g. acute, forensic, residential dementia care) |  |
| Population |  |
| Participants details (number, age, sex and diagnosis) |  |
| Study design |  |
| Study procedure |  |
| Intervention (if relevant)  Include:   - type of programme e.g. PA delivered or PA promoted; group or individual - mode, intensity, frequency and duration of PA - provider - delivery mode (F2F, unsupervised, remotely-supervised) - other key components (e.g., resources, sign-posting) |  |
| Comparison (if relevant)   - Potential options: no intervention, other PA intervention (specify), other non-PA intervention (specify) |  |
| Outcomes/measures (include method of collection, e.g. self-report, observation, device-based, mixed) |  |
| Key findings (intervention effects, data on correlates of physical activity) |  |
| Research recommendations/gaps in research specified |  |
